# Clinical judgement & evidence-based medicine: time for reconciliation

**DOI:** 10.4103/0971-5916.73418

**Published:** 2010-11

**Authors:** Ganesan Karthikeyan, Prem Pais

**Affiliations:** Department of Cardiology, All India Institute of Medical Sciences, New Delhi, India; * *Department of Medicine, St. Johns Medical College, Bangalore, India*

**Keywords:** Biases, clinical judgement, evidence-based medicine, heuristics

## Abstract

There is a popular perception that clinical judgement and evidence-based medicine are at loggerheads with each other. We examine the concepts of evidence and judgment as applied to clinical practice, and attempt to understand the reasons behind this imaginary divide.

Clinicians in the traditional mold, and more recently trained physicians who subscribe to the tenets of evidence-based medicine (EBM), often hold diametrically opposite viewpoints when making patient management decisions. These differences of opinion often appear irreconcilable and cause considerable animosity between otherwise well-intentioned professionals. Arguments are designed to pit the nebulous notion of “clinical judgment” against a loosely defined concept of EBM. In scientific meetings and teaching sessions at academic institutions where these exchanges most often occur, these discussions provide for much entertainment by emphasizing extreme and unrealistic scenarios, but contribute little towards offering any insight or clarification. An appreciation of the true meaning of what both “clinical judgment” and “EBM” stand for is essential to understanding, and hopefully, resolving this unpleasant standoff. The divide between “clinical judgment” and “EBM” is in fact nonexistent and is the result of a lack of understanding of what these terms actually represent^1^.

## What is clinical judgement?

The term clinical judgment conjures up visions of the archetypal clinician endowed with infinite wisdom and breathtaking clairvoyance. Flamboyance is another trait that readily comes to mind. In short, in popular conception, clinical judgment seems to be more about the clinician than about judgment. Fortunately for all of us, and our patients, clinical judgment is much more than that. For purposes of description, it can be considered the sum total of all the cognitive processes involved in clinical decision making. It involves the appropriate application of knowledge and individual expertise to the problem at hand. It would appear that this view of clinical judgment does not conflict with the tenets of EBM. But the problem arises (as we shall see later) because of the differing values attached to the different components of this cognitive process.

## What is EBM?

Sackett and colleagues^2^ describe EBM as the “conscientious, explicit, and judicious use of current best evidence in making decisions about the care of individual patients,…..integrating individual clinical expertise with the best available external clinical evidence from systematic research”. Contrary to popular belief, it is not about slavish adherence to external evidence or mindless extrapolation of trial results to the clinical setting. An essential component of the evidence-based decision making process is the ability of the clinician to comprehend the nature and strength of evidence and appropriately apply it to individual patients in his or her care. This ability to objectively appraise the available external evidence in the context of individual patients is in fact what clinical judgment is all about. Clinical judgment, as we see it, is therefore, a key component of EBM.

## The nature and evolution of evidence and the rationale for EBM

As some critics have pointed out, EBM is indeed “old hat”^3^. It has been around since the time of the first clinicians. These clinicians applied the best evidence available to them, in the treatment of their patients. What has changed is the nature of “evidence” itself. And it has changed fundamentally. Clinicians of yore drew upon their personal experiences, which in some cases were extensive, for evidence to support their practice. In those times, individual physician experience was often the largest and by far the easiest source of the available evidence. However, with the exponential growth in medical knowledge and technology, there is a large body of easily accessible, good quality evidence, which is incomparably larger than any individual clinician’s experience. More importantly, the quality of evidence from these two sources is fundamentally different.

An individual’s experience is inevitably coloured by his or her biases and preconceptions. More specifically, behavioural psychologists have shown that people rely on a limited set of heuristics to reduce the complex task of assessing probabilities and predicting values, to simpler judgmental operations^4^. These heuristics, by nature, are unreliable and result in systematic, and sometimes, severe biases. For instance, when a clinician sets out to prescribe a treatment to a patient, based on his experience with the particular treatment, he is likely to be influenced by the results in a similar patient he had previously treated (the representativeness heuristic), and any (easily recalled) dramatic results with the treatment (the availability heuristic). To complicate matters further, because of increasingly effective therapies, the magnitude of benefit (or harm) with any newer treatment is likely to be moderate at best^5^. It is impossible for any individual, however astute, to be able to discern a difference of this magnitude from random, temporally scattered experience. High quality external evidence is, therefore, required so that we do not miss the woods for the trees.

## Cause for conflict

Numerous “limitations” of EBM have been cited in the literature. As discussed by Strauss & McAlister^6^, much of this criticism arises out of misperceptions or misrepresentations of the basic principles of EBM. The most important issues which are at the heart of the conflict between EBM and its detractors are listed in the Table.. It is apparent that these are misconceptions rather than true limitations. The issue of blind adherence to algorithms is one false notion that has been alluded to earlier. It cannot be overemphasized that EBM does not advocate the indiscriminate application of evidence driven by blind adherence to guidelines and algorithms. We discuss the remaining issues here.

**Table.** Issues at the heart of the conflict between EBM and its detractors (with specific reference to the practice of medicine in India)

Commonly held misconceptions about EBM
Denigrates clinical judgment (and the clinician) Does not apply to care of individual patients Advocates a slavish, “cook-book” approach to treatment Ignores patient’s values and preferences

EBM is evidence-based medicine

### I. Evidence-based medicine denigrates clinical judgment (and by proxy, the clinician)

This is one of the biggest stumbling blocks in the widespread acceptance of EBM in countries like India, where the practice of medicine is still paternalistic and physician-centered. The central role of clinical judgment and expertise in decision-making, within the framework of EBM, has been pointed out earlier. Unfortunately, some (not all) clinicians differ from EBM adherents in their understanding of what “clinical expertise” entails. While EBM requires that the clinician objectively appraise the strength of evidence and make a decision about its applicability in a given context, some clinicians continue to persist with subjective, “black-box” methods for decision-making (“I feel this patient will do better with treatment A than treatment B”). Such methods have contributed to and propagated the mystique and allure of the erstwhile notion of “clinical judgment”. Such decisions are invariably biased and can affect the quality of care. All clinicians have a responsibility to make decision-making process explicit and open to critical appraisal. Viewing this as a curtailment of clinical freedom does justice neither to patient care nor to medical education.

### II. Evidence-based medicine does not apply to the care of individual patients

This is the most pervasive misconception in the way of widespread acceptance of EBM. As argued by Strauss & McAlister^6^, the universality of biologic variation makes the application of findings to individual patients problematic, whether these findings are from basic or applied research. Therefore this problem is not unique to EBM. Moreover, this issue cannot be resolved by clinical judgment either: short of performing an “n of 1” trial on all patients for all potential treatments (which is impractical), there is no way of definitely knowing the response of an individual patient. Till such time that personalized medicine, based on genomic and other biologic characteristics, becomes practical, EBM provides us with the best tools to individualized patient care. From its inception, EBM scholars have advocated the design and performance of large pragmatic trials with patient-important outcomes and have provided guidelines to apply the results to individual patients^5^,^7^,^8^. Judicious use of subgroup analyses also helps tailor treatment to individual patients^7^. For example, the use of an early, routine invasive strategy in the management of acute coronary syndromes significantly reduces the occurrence of a composite of death or myocardial infarction (MI)^9^. But, on analysis by troponin positivity, it has been shown that there is no benefit among patients who are troponin negative^9^. Another example is the differential benefits of coronary artery bypass graft (CABG) surgery in patients with coronary artery disease. A meta-analysis of randomized trials comparing CABG and medical therapy showed that CABG improved survival compared to medical therapy^10^. On subgroup analysis, it was found that this benefit was most pronounced in patients with left main coronary artery disease and triple vessel disease. Patients with single vessel disease did not derive any benefit^10^.

### III. Evidence-based medicine ignores patient values and preferences

EBM advocates the integration of the best external evidence, appraised by the discerning clinician, with patient values and preferences. But unfortunately, even practitioners of EBM have possibly valued the strength of evidence ahead of patient preferences. In an attempt to address this issue, the Grades of Recommendation, Assessment, Development, and Evaluation (GRADE) Working Group^11^ has developed a system, which separates the grading of the strength of recommendations from grading the quality of evidence. In this scheme, the strengths of recommendations are decided based on the balance between benefits and downsides, explicitly taking into account patient values. The GRADE system has two components. First, an assessment of the quality of evidence (which is graded as high, moderate, low or very low), and second the strength of recommendation (which may be strong or weak). Ordinarily the quality of evidence would have to be high or moderate for a strong recommendation to be made, and low or very low quality of evidence would result in a weak recommendation. But the recommendation can be modulated to accommodate patient values, preferences and perhaps even social and economic considerations. For example, although there is high quality evidence favouring primary angioplasty for acute ST elevation MI, in the context of the average Indian patient, guideline formulators would probably only make a weak recommendation. Several clinical societies have endorsed and adopted the GRADE system for formulating clinical practice guidelines^12^

## Application of evidence to individual patients

Application of evidence to individual patient management is such a contentious issue that it deserves further elaboration. Once the clinician has located the evidence relevant to the patient’s clinical condition, he/she needs to decide about its applicability. Measures of treatment effectiveness obtained from clinical trials are average measures and due to the inevitable biologic variability, are bound to vary across the population. But it pays to keep in mind that patients enrolled in clinical trials are likely to be much more similar to each other than they are likely to be distinct. As a result, major differences in the magnitude of effect are unlikely. Qualitatively different effects (harm for some and benefit for others) are extremely rare. Therefore, the results of clinical trials can be applied at the bedside, to patients broadly similar to those in clinical trials with the anticipation of benefits similar to that seen in the trials. The presence of co-morbidity and large differences in age from the study population are some factors, which can legitimately influence the clinician’s decision.

### Subgroup analyses

A related area of relevance to individual-patient decision-making is the use of subgroup analyses. As clinicians, the results of subgroup analyses hold intuitive appeal to us. It is sobering to remember that, embedded in any clinical trial population, there are an infinite number of subgroups and “subgroup effects”, most of which are spurious. The real difficulty is in seeking out the true subgroup effects. In evaluating subgroup analyses, the following issues need to be considered: *(i)* were the analyses prespecified or were they embarked upon after “looking” at the data, *(ii)* how large are the effects? *(iii)* is the subgroup effect biologically plausible?, *(iv)* is it statistically different from the rest of the study population?, and finally *(v)* is there corroborative evidence from other studies? The criteria for accepting subgroup results need to be stringent because, as we pointed out, most are spurious^13^and indeed, very few subgroup analyses have stood the test of time.

## A place for evidence and a place for judgement

We have tried to show that the clinician has a central role to play in the scheme of evidence-based delivery of care. EBM only requires that the clinician be sufficiently familiar with the evidence-base in his/her field and be able to objectively appraise it, so that he or she can apply it appropriately in practice. Clinicians should acknowledge that EBM is an important phase in the evolution of the practice of medicine, which attempts to deliver care of uniformly high quality. As the principal agents responsible for delivering this care, they should educate and equip themselves better for this vital role.

## References

[1] Karthikeyan G (2007). Evidence-based medicine and clinical judgment: an imaginary divide. *J Am Coll Cardiol*.

[2] Sackett DL, Rosenberg WMC, Gray JAM, Haynes RB, Richardson WS (1996). Evidence based medicine: what it is and what it isn’t. *BMJ*.

[3] Grahame-Smith D (1995). Evidence based medicine: Socratic dissent. *BMJ*.

[4] Tversky A, Kahneman D (1974). Judgment under uncertainty: Heuristics and biases. *Science*.

[5] Yusuf S, Collins R, Peto R (1984). Why do we need some large, simple randomized trials?. *Stat Med*.

[6] Straus SE, McAlister FA (2000). Evidence-based medicine: a commentary on common criticisms. *CMAJ*.

[7] Guyatt GH (2007). Evidence-based decision-making is individualized clinical decision-making. *Chin J Evid-based Med*.

[8] Guyatt GH, Haynes RB, Jaeschke RZ, Cook DJ, Green L, Naylor CD (2000). Users’ guides to the medical literature XXV. Evidence-Based Medicine: Principles for applying the users’ guides to patient care. Evidence-based Medicine Working Group. *JAMA*.

[9] Mehta SR, Cannon CP, Fox KAA Wallentin L, Boden WE, Spacek R (2005). Routine vs selective invasive strategies in patients with acute coronary syndromes: a collaborative meta-analysis of randomized trials. *JAMA*.

[10] Yusuf S, Zucker D, Peduzzi P, Fisher LD, Takaro T, Kemedy JW (1994). Effect of coronary artery bypass graft surgery on survival: overview of 10-year results from randomised trials by the Coronary Artery Bypass Graft Surgery Trialists Collaboration. *Lancet*.

[11] Atkins D, Best D, Briss PA, Eccles M, Falck-Ytter Y, Flottorp S (2004). Grading quality of evidence and strength of recommendations. *BMJ*.

[12] Schünemann HJ, Jaeschke R, Cook DJ, Bria WF, El-Solh AA, Ernst A (2006). ATS Documents Development and Implementation Committee. An official ATS statement: grading the quality of evidence and strength of recommendations in ATS guidelines and recommendations. *Am J Respir Crit Care Med*.

[13] Yusuf S, Wittes J, Probstfield J, Tyroler HA (1991). Analysis and interpretation of treatment effects in subgroups of patients in randomized clinical trials. *JAMA*.

